# Suspected gut barrier disruptors and development of food allergy: Adjuvant effects and early immune responses

**DOI:** 10.3389/falgy.2022.1029125

**Published:** 2022-11-22

**Authors:** Elena Klåpbakken Drønen, Ellen Namork, Hubert Dirven, Unni Cecilie Nygaard

**Affiliations:** ^1^ Department for Chemical Toxicology, Division for Climate and Health, Norwegian Institute of Public Health, Oslo, Norway; ^2^ Section for Immunology, Division for Infection Control, Norwegian Institute of Public Health, Oslo, Norway

**Keywords:** barrier disruptors, food allergy, peanut, oral, cholera toxin, deoxynivalenol, glyphosate, house dust mite

## Abstract

Food allergy is an increasing public health challenge worldwide. It has recently been hypothesized that the increase in exposure to intestinal epithelial barrier-damaging biological and chemical agents contribute to this development. In animal models, exposure to adjuvants with a food allergen has been shown to promote sensitization and development of food allergy, and barrier disrupting capacities have been suggested to be one mechanism of adjuvant action. Here, we investigated how gut barrier disrupting compounds affected food allergy development in a mouse model of peanut allergy. Sensitization and clinical peanut allergy in C3H/HEOuJ mice were assessed after repeated oral exposure to peanut extract together with cholera toxin (CT; positive control), the mycotoxin deoxynivalenol (DON), house dust mite (HDM) or the pesticide glyphosate (GLY). In addition, we investigated early effects 4 to 48 h after a single exposure to the compounds by assessing markers of intestinal barrier permeability, alarmin production, intestinal epithelial responses, and local immune responses. CT and DON exerted adjuvant effects on peanut allergy development assessed as clinical anaphylaxis in mice. Early markers were affected only by DON, observed as increased IL-33 (interleukin 33) and thymic stromal lymphopoietin (TSLP) alarmin production in intestines and IL-33 receptor ST2 in serum. DON also induced an inflammatory immune response in lymph node cells stimulated with lipopolysaccharide (LPS). HDM and GLY did not clearly promote clinical food allergy and affected few of the early markers at the doses tested. In conclusion, oral exposure to CT and DON promoted development of clinical anaphylaxis in the peanut allergy mouse model. DON, but not CT, affected the early markers measured in this study, indicating that DON and CT have different modes of action at the early stages of peanut sensitization.

## 1 Introduction

The prevalence of food allergies appears to have increased in the western world over the past decades and is considered a major public health concern with considerable costs (1, 2) Although the reason for this increase is largely unknown, environmental exposures and lifestyle factors have been suggested as possible causes in addition to genetical susceptibility (3). Biological agents or chemical contaminants with adjuvant capacities may promote food allergy development through modulating and/or enhancing the immune response towards antigens present in food (4).

In animal models, exposure to adjuvants together with food allergens has been shown to promote, and to be necessary, for sensitization and development of food allergy (5–7). The most used adjuvant in these models, cholera toxin (CT), has also been shown to have gut barrier disrupting properties (8). Gut barrier disruptors are compounds capable of activating or damaging the intestinal epithelial barrier. This may lead to a leaky gut with increased uptake of allergens and other compounds from the intestinal lumen, into blood, resulting in more direct contact with the immune system. Furthermore, a damaged epithelial barrier may elicit responses resulting in production of endogenous danger signals such as alarmins. The cytokines interleukin 33 (IL-33), thymic stromal lymphopoietin (TSLP) and IL-25 act as alarmins released due to cell damage caused by infection or other cellular stress (9, 10). These alarmins play important roles in maintaining gut homeostasis but can also stimulate a pro-allergic microenvironment by typically activating T helper 2 and type 2 innate lymphoid cells (9–16).

A number of biological and chemical insults from the environment have been shown to impair mucosal barrier integrity and function [reviewed in ref. (17)]. These include the common mycotoxin deoxynivalenol (DON) as present in grains (18) and house dust mite (HDM) extracts (19, 20) which also are promoting development of food and airway allergy, respectively, in animal models (7, 21). The proteolytic HDM allergen Der p1 has been detected in human gut and been demonstrated to impair epithelial barrier both *in vitro* (human colonic biopsies) and *in vivo* (mice) (19). Presence of HDM allergen in breast milk is associated with an increased risk of atopic sensitization and respiratory allergy in children (22). Glyphosate (GLY) has been reported to have effects on alarmin production (23) and effects on gut microbiota (24), but has unknown barrier disrupting capacities. Thus, we hypothesized that compounds able to affect the gut barrier integrity and function play a role in promoting development of food allergy. Our working hypothesis was that compounds with allergy adjuvant potential induce changes in barrier function early after exposure, and that markers for early changes could potentially be used to identify adjuvants promoting development of food allergy.

The objective of the present study, therefore, was to investigate how these known and suspected gut barrier disruptors affected food allergy development in a well-established mouse model of peanut allergy. Sensitization and clinical peanut allergy development were assessed after repeated oral exposures to peanut extract together with one of the barrier disrupting compounds CT, DON and HDM, or the suspected barrier disruptor GLY. Additionally, early effects of a single oral exposure to the compounds were investigated, including markers of intestinal integrity, alarmin production and local immune responses 4 to 48 hours after exposure. The present study is contributing knowledge to the concept of “the epithelial barrier hypothesis” (25) and proposes that the increase in epithelial barrier-damaging agents linked to industrialization, modern lifestyle and climate change contribute to the rise in allergic, autoimmune and other chronic conditions.

## 2 Materials and methods

**Figure 1:**
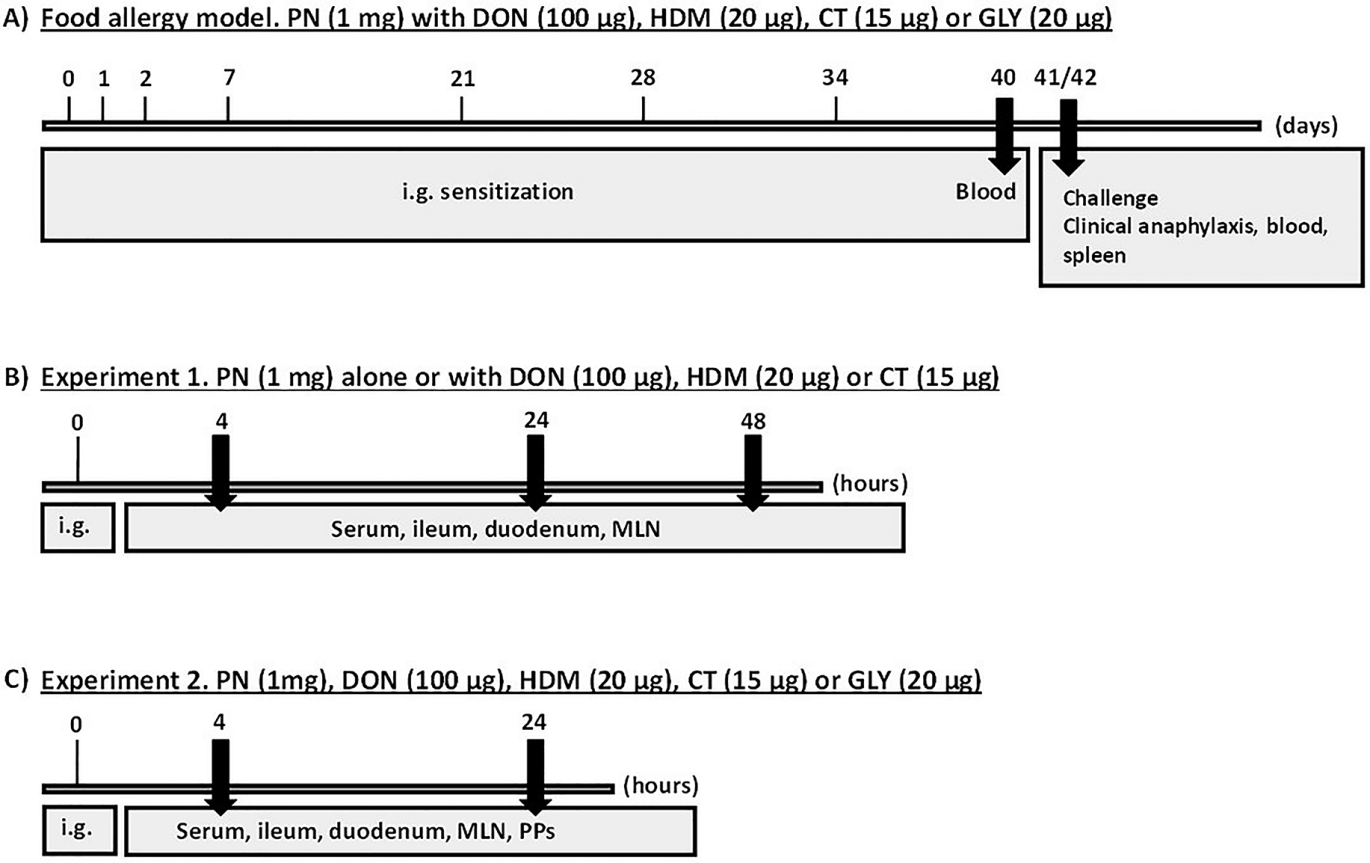
Experimental scheme. (**A**) Food allergy model. Mice were sensitized by repeated i.g. exposure to PN with a barrier disruptor up to 34 days and challenged with a high dose of PN given i.p. at day 41 or 42 to assess food allergy development. Clinical anaphylaxis was assessed, before collection of blood and spleen. (**B,C**) Short-term experiments. Mice were exposed once i.g. to a barrier disruptor with or without PN in experiments 1 and 2 to assess early effects. Blood and tissues were collected at 4, 24 and 48 (**B**) and 4 and 24 (**C**) hours after exposure.

The experimental setup is illustrated in Figure 1. The allergy-promoting capacity of the selected barrier disruptors was assessed in a food allergy mouse model where mice were repeatedly exposed to the selected barrier disruptors together with peanut extract (PN), and thereafter challenged with a high PN dose to assess clinical anaphylaxis. Two short-term mouse experiments were performed to investigate the early effects (4, 24 and 48 h) on the gut barrier after a single exposure of the barrier disruptors with or without PN. The studies were approved by the Experimental Animal Board under the Ministry of Agriculture in Norway (FOTS ID 11121). The experiments were performed in conformity with the laws and regulations for experiments with live animals in Norway.

### 2.1 Test solutions

Cholera toxin (CT), deoxyvalenol (DON) and house dust mite (HDM) were defined as known barrier disruptors (7, 8, 21), and the pesticide Glyphosate (GLY) as a suspected barrier disruptor. The doses for CT (EMD Biosciences Inc., San Diego, CA, United States), DON (Sigma Aldrich, St. Louis, MO, United States), HDM (Stallergenes Greer, London, UK), and GLY (SUPELCO, Bellefonte, PA, United States), were based on literature where exposure to these doses gave biological effects (doses and references are stated in Table 1). PBS (10 mM 0.9% NaCl pH 7.4) was used as negative control and as vehicle. Peanut extract (PN, Stallergenes Greer, London, UK) was used as the food allergen.

**Table 1:** Test solutions, doses (all oral exposures) and literature used to choose dose levels.

	Test compounds and dose per animal (in 250 µl PBS)			Effects demonstrated at this dose level in earlier studies
	exp. 1^†^	exp. 2^†^	Food allergy model^‡^	
PBS: vehicle ctr.	PBS	PBS	PBS	–
PN wo adjuvant: neg. ctr.	1 mg PN	1 mg PN	1 mg PN	Peanut food allergy (with CT) (5)
CT: pos. adjuvant ctr.	15 µg CT	15 µg CT	15 µg CT	Increased food allergy (7)
	1 mg PE		1 mg PN	
DON	100 µg DON	100 µg DON	100 µg DON	Increased food allergy (7)
	1 mg PN		1 mg PN	
HDM	20 µg HDM	20 µg HDM	20 µg HDM	Increased colonic permeability (19)
	1 mg PN		1 mg PN	
GLY		20 µg GLY	20 µg GLY	Effects on gut microbiome (26)
			1 mg PN	Effects of Roundup®^§^ (27)
				Effects on behavior (24, 28)
CT wo allergen: neg. ctr.			15 µg CT	Neg. ctr.

Pos, positive; Neg, negative; ctr, control; wo, without.

^†^ Single exposure.

^‡^ Repeated exposures.

^§^ Roundup® herbicide contains approximately 40% GLY (Monsanto Company, St. Louis, MO, United States).

### 2.2 Animals

Female C3H/HeOuJ mice were purchased from Charles River Laboratories (Sulzfeld, Germany), and Jackson Laboratory (Bar Harbor, ME, United States) for exp. 2 due to supply issues. The C3H/HeJ mouse strain is commonly used in food allergy models (7, 29). The strain is a high IgE responder and is prone to develop anaphylaxis and is therefore well suited as a model animal for food allergy development (30, 31).

The mice were 4 to 5 weeks old at arrival and randomly allocated in groups and housed on Nestpack bedding (Datesand Ltd, Manchester, UK) at room temperature of 21 ± 2 °C, humidity of 55 ± 10% and exposed to 12 h light/dark cycle. The mice were given pelleted feed (RM1, SDS, Essex, UK) and tap water *ad libitum*, and were acclimatized for at least one week before dosing. The experiments with live animals were performed in conformity with the laws and regulations in Norway and were approved by the Experimental Animal Board under the Ministry of Agriculture in Norway (FOTS ID 11121).

### 2.3 Food allergy model

#### 2.3.1 Immunization, challenge, and anaphylaxis assessment

The present peanut food allergy model was based on the mouse model of lupine allergy developed by Vinje et al. (6), and the mouse model of peanut anaphylaxis developed by Li et al. (5). According to Figure 1A and Table 1, twelve mice in each of the 7 groups were immunized with intragastric (i.g.) gavage at day 0, 1, 2, 7, 21, 28 and 35 with PN alone or mixed with the different barrier disruptors. CT with PN was used as a positive control of food allergy development as CT has a strong adjuvant effect (29). In order to limit the number of animals used, control groups were limited to the vehicle-exposed (PBS) and non-sensitized mice (PN or the positive control CT alone).

A challenge of a high dose of PN was given intraperitoneally (i.p). at the end of the experiment to induce anaphylaxis (6), and thereby to confirm clinically relevant signs of food allergy. This challenge was given i.p. since this was previously demonstrated to give more robust responses compared to oral challenge (32).

Due to practical considerations, half of the mice in each group were challenged at day 41 and 42, respectively. Prior to challenge, the mice were weighed, 100 µl blood sampled from the vena saphena in Microvette tubes without heparin (Sarstedt, Nümbrecht, Germany) and serum stored at −20 °C for detection of Ara h2 specific IgE (see below). The mice were challenged with an i.p. injection of 5 mg PN in 250 µl of PBS (5). Due to dilution errors on one of the days, challenge with 2.5 mg PN in 250 µl PBS was given to approximately half of each of the groups of mice. Temperature measurements (at 0, 15 and 30 min) and clinical anaphylaxis score were recorded and reported as previously described (33). Scores: 0 – no symptoms; 1 – scratching and rubbing around the nose and head; 2 – puffiness around the eyes and mouth, diarrhea, pili erecti, reduced activity, and/or decreased activity with an increased respiratory rate; 3 – wheezing, laboured respiration, cyanosis around the mouth and tail; 4 – no activity after prodding or tremor and convulsion; 5 – death. In case of strong anaphylactic responses (grade 4), animals were exsanguinated immediately. After 30 min, all challenged mice were exsanguinated, and serum was collected. Due to anaphylaxis, we did not manage to collect serum from four and two mice in the PN + CT and GLY + PN groups, respectively. Serum was prepared and stored at −20 °C until detection of total IgE and the anaphylaxis marker mMCP-1 (see below). Spleens were collected and kept in HBSS containing 2% fetal bovine serum and 1% penicillin/streptomycin on ice.

Levels of serum mMCP-1 after challenge were assessed as marker for intestinal anaphylaxis responses (34). Serum concentrations were determined using mouse CPT-1 (mMCP-1) ELISA kit according to the manufacturer's protocol (ELISA Ready-SET-Go! ® Invitrogen by Thermo Fisher).

#### 2.3.2 Spleen cell preparation, PN stimulation and detection of released cytokines

Compared to lymph nodes, spleen yields an abundant cell number and were therefore chosen to assess expression of cytokines reflecting systemic changes to the immune system reported in food allergy models (5, 6). Spleen cells were prepared, stimulated, and collected as described by Vinje et al. (35), using PN at a final concentration of 1 mg/ml to assess the cytokine responses induced mainly in allergen-specific cells.

The cytokines TNF-α, INF-γ, IL-1β, IL-2, IL-6, IL-10, IL-13, and IL-17 were detected in cell supernatants using BD Cytometric bead array (CBA) Mouse Soluble Protein Flex Sets, measured on a BD LSR II flow cytometer. The cytokines were analyzed using the FCAP Array software (BD Biosciences, San Jose, CA, United States) according to the manufacturer's protocol.

#### 2.3.3 Measures of peanut sensitization – total IgE and Ara h2-specific IgE

As described for other food allergen extracts (33), development of ELISAs for PN-specific IgE was challenging (unpublished data) due to high background signals. Therefore, as predictors of peanut allergy sensitization, the levels of the peanut component Ara h2 specific IgE and total IgE in serum were analyzed. IgE specific to the most important peanut allergen Ara h2 is considered a reliable marker of severe peanut allergy in humans (36, 37). Total IgE are reflecting specific IgE in animal models with controlled allergen exposures (33, 38).

Ara h2-specific IgE was assessed in serum samples collected immediately prior to challenge, since specific IgE passes from the blood to the tissues during anaphylaxis. The ELISA assay was developed in-house. 100 µl of monoclonal rat anti-mouse IgE (2 µg/ml, Experimental Immunology unit, University of Louvain, Brussels, Belgium) in 0.05 M bicarbonate buffer (pH 9.6) was added in each well plate (Nunc MaxiSorpTM flat-bottom 96 well plate, Thermo Fisher Scientific) and incubated 1 h at 21 °C following an over-night incubation at 4 °C. The plates were then washed in TBS/Tween (50 mM Tris/HCL-buffer pH 8.0 with 0.05% Tween 20) using an automatic plate washer (405 LS microplate washer, Biotek), and blocked using 4% BSA (Bovine Serum Albumin A7930-100G, Sigma Aldrich) in PBS (BSA/PBS) and incubated for 1 h at 21 °C. After washing, test serum from the experiments were added, and serial dilutions of a mouse serum pool containing anti-Ara h2 from PN-immunized mice (The Norwegian Institute of Public Health) was added as doublets for standard curve generation. After washing, 100 µl of biotin-labeled Ara h2 (3 µg/ml, Biotin Natural Ara h2, B1-AH2-1, Indoor Biotechnologies) in 4% BSA in PBS was added, and again incubated and washed as above. Poly-HRP-strepatividin (Poly-HRP-strepatividin N200, Thermo Fisher Scientific) was diluted 1:40,000 in 4% BSA/PBS and 100 µl added to each well. Plates were then incubated and washed, before addition of 100 µl of TMB solution (TMB Stabilized Chromogen SB01, Life Technologies EuroPN B.V., Bleiswijk, Netherlands) and incubated at RT for 15 min. The reaction was stopped by 50 µl of sulfuric acid 95%–97% (Merck Milipore, Burlington, MA, United States) in each well. Plates were read at 450 nm in a BioTek microplate-reader.

Serum levels of the allergic sensitization marker total IgE was determined using IgE Mouse uncoated ELISA kit according to the manufacturer's protocol (Invitrogen by Thermo Fisher Scientific). This was assessed in serum collected after challenge and exsanguination due to limited amounts of serum from the sample collected before challenge.

### 2.4 Short-term experiments

Intestinal epithelial responses were measured as release of the alarmins IL-33 and its receptor ST2, TSLP and IL-25 in intestinal tissue homogenate supernatants from duodenum and ileum. ST2 in serum was also a marker of altered barrier function (11, 39). Serum levels of fatty acid-binding protein 2 (FABP2) and the peanut component Ara h2 were also analyzed in serum as markers of gut barrier permeability (40). The lymphoid responses were measured as release of the cytokines TNF-α, IFNγ, IL-1β, IL-2, IL-4, IL-5, IL-6, IL-10, IL-13, and IL-17 from cells in the mesenteric lymph nodes (MLNs), Peyer's patches (PP) or spleen. As 4–48 h after exposure was regarded to be too early to detect allergen-specific recall responses, cells were stimulated with ConA to activate T cells or LPS to activate B cells and monocytes. To investigate the impact of PN presence, animals were exposed to PN together with the barrier disruptor in experiment 1, and only exposed to the barrier disruptor in experiment 2.

#### 2.4.1 Experiment 1: Early effects 4, 24 and 48 h after a single exposure to the test compounds with PN

Five groups of mice, 5 in each group, were exposed once by i.g. gavage to 250 µl of test solutions according to Table 1. At 4, 24 and 48 h after exposure, the mice were anesthetized with isoflurane gas administered as a 3.5% mixture with medical O_2_ in a coaxially ventilated open mask and exsanguinated by heart puncture before cervical dislocation. The collected blood samples were kept for 1–3 h at RT before the samples were prepared and stored at −80 °C until further use.

The mesenteric lymph nodes (MLNs) were excised and kept on ice in 5 ml Meinecker tubes with 1 ml HBSS (Hanks' Balanced Salt Solution, Gibco® by Thermo Fisher Scientific, Waltham, MA, United States) containing 2% fetal bovine serum (FBS superior, Biochrom, Cambridge, UK) and 1% penicillin/streptomycin (10,000 units/ml penicillin, 10 mg/ml streptavidin, PAA Laboratories Inc, Etobicoke, ON, Canada), to ensure cell viability.

The small intestine was excised, and duodenum and ileum were placed on cold metal plates to minimize degradation of proteins. The duodenum was defined as the top 7 cm of the small intestine closest to the stomach and the ileum as the lower 7 cm closest to the caecum. Duodenum and ileum were flushed thoroughly with cold PBS using a syringe, and visceral adipose tissue was removed. Two 1 cm sections from each of the 7 cm segments were snap frozen in 1.8 ml cryo tubes in liquid nitrogen and stored at −80 °C until homogenization.

##### 2.4.1.1 Homogenization of intestinal tissue

Immediately after thawing the intestinal samples were weighed in 1.5 ml tubes (Micro Tubes, Brand Scientific Equipment Pvt. Ltd., Wertheim, Germany). and 0.6 ml of RIPA lysis buffer with 0.04% protease-inhibitor was added to the tubes to avoid the tissue from drying and to reduce protein degradation (7).

The tissue samples were transferred to 2 ml CK28-R tubes containing ceramic beads (Bertin Instruments) and homogenized using Precellys 24 tissue homogenizer (Bertin Instruments) at 6000 rpm (2 × 30 s, 30 s pause). After homogenization, the tubes were left to cool on ice for up to 30 min to diminish frothing. Samples were centrifuged at 1970 × g for 5 min, and the supernatants collected and stored at −80 °C until analysis.

##### 2.4.1.2 ELISA analysis

Serum samples or tissue homogenate supernatants were analyzed using ELISA kits according to the manufacturer's protocol: TSLP, IL-25, IL-33, and the IL-33 receptor ST2 (Invitrogen, Thermo Fisher Scientific), FABP2 (MyBioSource, San Diego, CA, United States) and Ara h2 (Indoor biotechnologies, Charlottesville, VA, United States). Ara h2 concentrations were determined using an anti-peroxidase-conjugated Fab fragment for detection (Jackson ImmunoResearch Laboratories Inc., West Grove, PA, United States). Optical density (OD) values were measured using a Microplate Reader (Elx808 Absorbance Reader, BioTek, Shoreline, WA, United States) with software Gen5TM (BioTek). Protein concentrations were determined based on a standard curve provided in the kits. For results from the intestinal homogenates, all concentrations were normalized by dividing by the respective tissue weight.

##### 2.4.1.3 Lymph node cell preparation, stimulation, and determination of cytokine release

Preparation of MLNs was performed as described for spleen cells. Since the early timepoints are too early for investigating allergen-specific responses, unspecific stimuli were used to assess cellular immune function (41, 42). MLN cells were stimulated for 48 h with either 5 µg/ml ConA (Concanavalin A from Canavalia ensiformis, Sigma-Aldrich) for T cell stimulation, or 10 µg/ml LPS (lipopolysaccharides from Escherichia coli 026: B6, Sigma-Aldrich) for B cell and monocyte stimulation, or culture medium alone. Cell supernatants were collected and stored, and cytokine concentrations of TNF-α, IFNγ, IL-1β, IL-5, IL-6, IL-10, IL-13 and IL-17 were determined as described in the food allergy model.

#### 2.4.2 Experiment 2: Effects 4 and 24 h after a single exposure to the test compounds

Mice were exposed to the same test-solutions as in exp. 1, as well as the pesticide GLY, but not co-exposed to PN (Table 1). All adjustments and changes in exp. 2 were based on results from exp. 1: time-points 4 and 24 h were chosen since few exposure-related changes were observed at 48 h, The number of mice was increased to 8 per group at each time-point. Furthermore, the whole length (7 cm) of intestinal tissue was used for homogenization. In addition to MLNs, up to seven PPs, primarily sampled from the ileum were collected, but also from the jejunum and duodenum if needed. PPs from each group were pooled, processed, and prepared as described for MLNs. Cytokine release from stimulated MLNs and PPs were determined as described for exp. 1. Levels of the cytokines IL-17, Il-10 and IL-5 were below the detection limit in exp. 1, and therefore excluded in exp. 2. Assessment of IL-2 and the Th2 cytokine IL-4 was included. FABP2, Ara h2 and IL-25 were excluded in exp. 2 due to low levels in both intestines and serum samples in exp.1. All ELISA analyzes in exp. 2 were incubated overnight on microwell plates at +4 °C to increase the sensitivity.

### 2.5 Statistical analysis

Non-parametric statistics were used as the assumptions of equal variance and a normality distribution in our data sets were violated for most of the endpoints. A Kruskal-Wallis test was initially performed, and if a *p*-value ≤ 0.05 was detected, we conducted a Dunn's multiple comparison test to identify which groups were different. Correlation of anaphylactic score and body temperature drop in the food allergy model was performed by Spearman rank-order correlation test. All analyzes were performed in GraphPad Prism version 7.00 for Windows (GraphPad Software, La Jolla, CA, United States).

## 3 Results

### 3.1 Food allergy development

#### 3.1.1 Anaphylaxis

**Figure 2:**
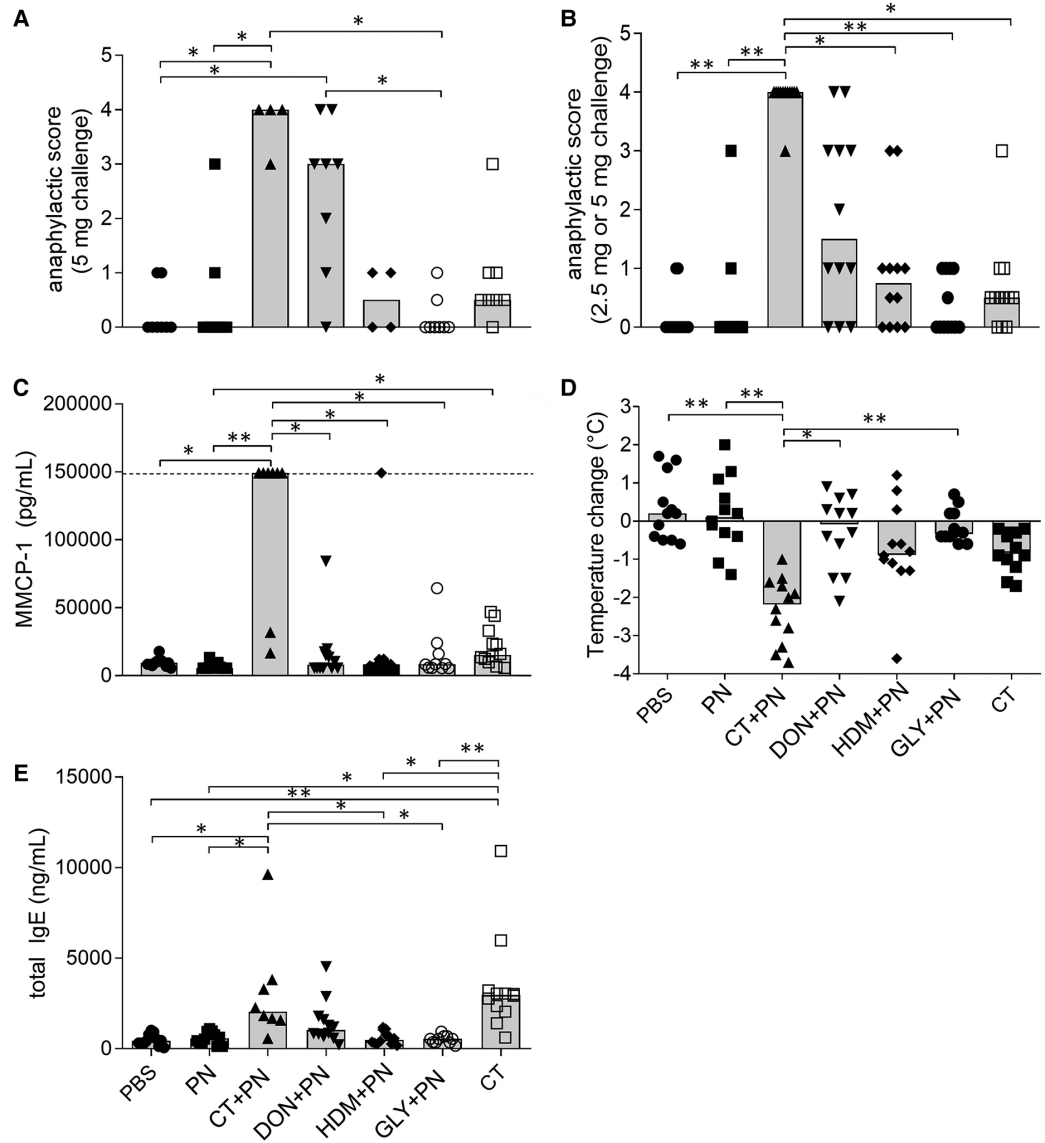
Anaphylaxis and sensitization after repeated immunizations with peanut extracts. (**A**) Anaphylactic score after i.p. challenge of 5 mg PN (*n* = 8 in PBS, PN, PN + DON, PN + GLY and CT groups, *n* = 4 in CT + PN and PN + HDM groups). (**B**) Anaphylactic score after i.p. challenge of 2.5 or 5 mg PN (*n* = 12). (**C**) Murine mast cell protease 1 (MMCP-1) concentrations in serum collected 30 min after PN challenge (PBS, PN, PN + DON, CT, all *n* = 12), PN + CT (*n* = 8), GLY + PN (*n* = 10). Dotted line represents the upper assay detection limit. (**D**) Temperature-change over the first 30 min after PN challenge (*n* = 12). (**E**) total IgE concentrations in serum collected after PN challenge (PBS, PN, PN + DON, CT, *n* = 12. PN + CT, *n* = 8. GLY + PN, *n* = 10). Stars denote statistically significant differences (*p < 0.05, **p < 0.001) between groups. Columns denote the group median value while each symbol denotes the individual animal.

After the PN challenge, clinical anaphylaxis was assessed as anaphylactic score, drop in rectal temperature and presence of mast cell protease 1 (mMCP-1) in serum. The anaphylactic scores for mice challenged with 5 mg PN were clearly higher in the groups immunized with DON + PN and CT + PN, reaching statistical significance compared to two (PBS and GLY + PN) and three (PBS, PN and GLY + PN) of the other groups, respectively (Figure 2A). When including both the 2.5 and 5 mg challenge doses in the statistical analysis (Figure 2B), thus doubling the number of data points per group, the CT + PN immunized group had significantly higher scores than all other groups except DON + PN. The increase in the DON + PN group did not reach statistical significance (Figure 2B), suggesting that the higher challenge dose of 5 mg PN was needed to elicit a clinical anaphylaxis score.

The rectal body temperature showed a strong decrease in the CT + PN group, reaching statistically significance compared to all other groups except the CT-group (Figure 2D). Four animals in the CT + PN group had strong anaphylactic responses (Figure 2D), and serum samples for analyzing mMCP-1 levels were not possible to collect from these animals. Still, the level of mMCP-1 in serum from the other animals in the CT + PN immunized group was statistically significantly higher compared to all other groups except for the CT-group (Figure 2C). A weak but statistically significantly elevated mMPC-1 level was also observed in the CT group, as compared to the PN group. The group exposed to CT only and the groups immunized with HDM + PN and GLY + PN did not affect the anaphylaxis endpoints.

#### 3.1.2 Sensitization

The levels of Ara h2 specific-IgE in serum before PN challenge were below the assay detection limit for most samples (data not shown). As expected for the positive control group, the levels of total IgE were statistically significantly increased in the CT + PN group compared to most groups, except for DON + PN and CT (Figure 2E). The CT group also reached statistically significantly higher levels of total IgE compared to all groups except CT + PN and DON + PN. The DON + PN group showed a (non-significant) tendency of increased total IgE levels in serum. The DON + PN group did not display statistically significantly lower total IgE levels compared to the CT and CT + PN groups (Figure 2E). As the levels of total IgE were measured in blood sampled after challenge with PN, the levels were most likely decreased because of the anaphylactic reaction (32, 43), supporting that sensitization was induced in the DON + PN group.

#### 3.1.3 Lymphoid immune cell responses

**Figure 3:**
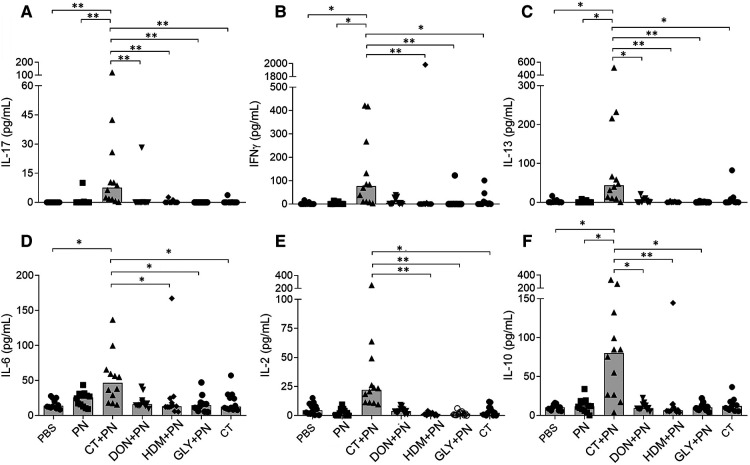
Cytokine release from PN-stimulated spleen cells ex vivo; **(A)** IL-17, **(B)** INFγ, **(C)** IL-13, **(D)** IL-6, **(E)** IL-2 and **(F)** IL-10. Mice were repeatedly immunized and received a PN challenge (*n* = 12), followed by spleen cell harvesting. Brackets denote statistically significant differences (*p < 0.05, **p < 0.001) between two groups. Columns denote the group median value while each symbol denote individual animals.

The lymphoid immune responses were assessed as release of the cytokines IL-1β, IL-2, IL-6, IL-10, IL-13, IL-17, IFNγ and TNF-α from spleen cells stimulated with PN ex vivo. As expected, the negative controls, PBS and PN without adjuvant, did not induce cytokine production. However, in positive control group immunized with CT + PN, all cytokines except IL-1β and TNF-α showed statistically significantly enhanced levels (Figure 3). A similar, but weak trend (statistically significantly different from the PBS group) was observed for TNF-α, while no group differences were observed for IL-1β (data not shown). Induction of Th2, but also Th1 and Th17 cytokines have been reported in previous food allergy models and may differ by mouse strain as well as type of adjuvant, antigen dose and affinity (6). None of the other groups showed significantly higher cytokine release after PN stimulation.

### 3.2 Short-term experiments

#### 3.2.1 Intestinal epithelial responses: Il-33, ST2, TSLP and Il-25 in duodenum and ileum

**Figure 4:**
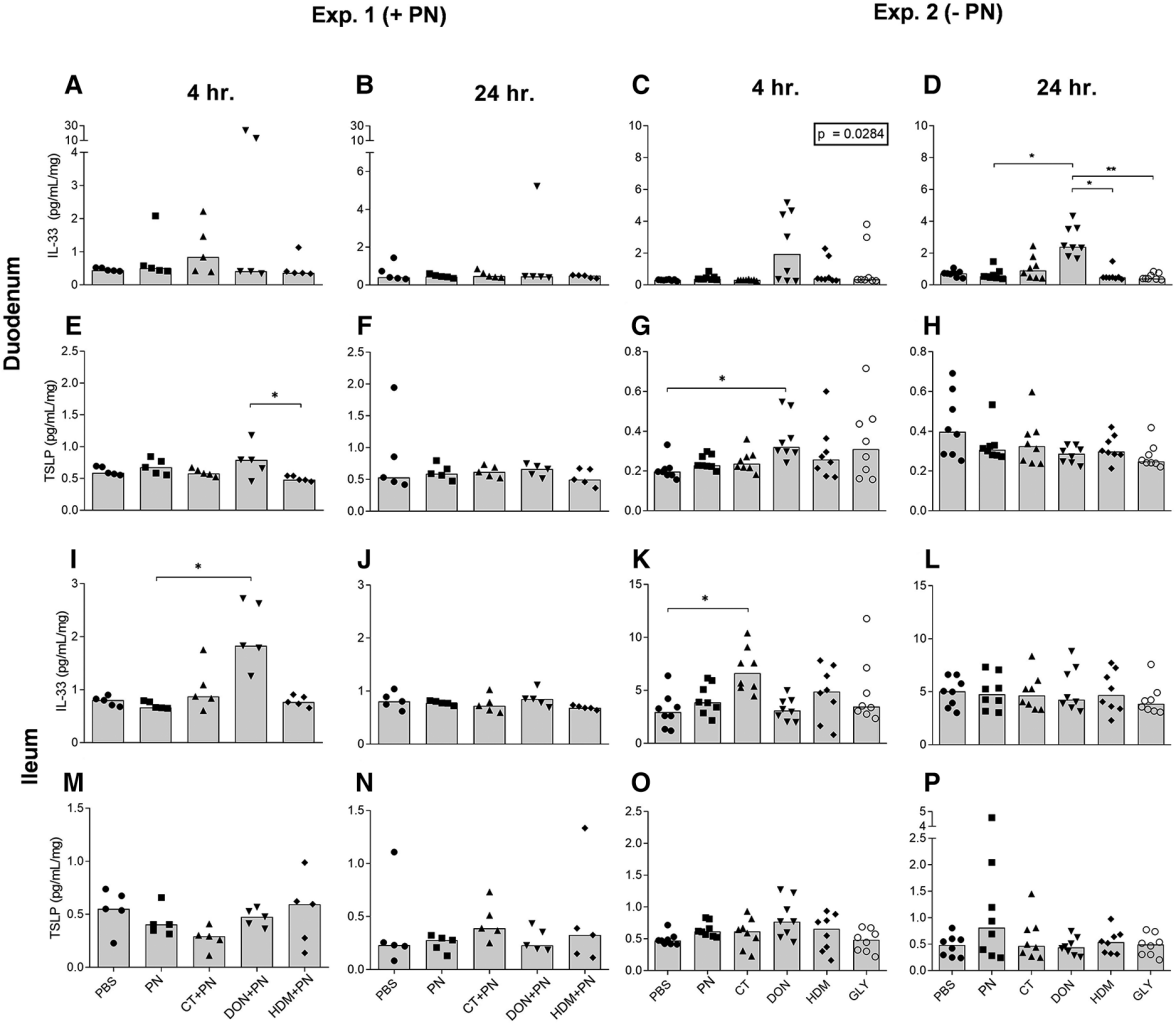
IL-33 and TSLP concentrations per mg tissue in duodenum and ileum after a single i.g. dose of PBS, PN or a barrier disruptor with (exp. 1, *n* = 5) or without (exp. 2, *n* = 8) PN. The figures show levels at 4 h **(A, E, I, M**) and 24 h **(B, F, J, N**) in exp. 1, and at 4 h (**C, G, K, O**) and 24 h (**D, H, L, P**) in exp. 2. (**A, B, C, D**) shows IL-33 and (**E, F, G, H**) shows TSLP in duodenum. (**I, J, K, L**) shows IL-33 and (**M, N, O, P**) shows TSLP in ileum. Brackets denote statistically significant differences (*p < 0.05, **p < 0.001) between two groups. Columns denote the group median value while each symbol denotes the individual animals. Inserted box: *p* value for the Kruskal-Wallis test.

IL-33 levels were increased in the DON exposed group compared to the other groups after 4 and 24 h, although only statistically significant at 24 h in duodenum in exp. 2 without PN (Figure 4D) and at 4 h in ileum exp. 1 with PN (Figure 4I). Exposure to CT alone only statistically significantly affected the level of IL-33 in ileum after 4 h in exp. 2 (Figure 4K). For the other compounds tested, no statistically significant differences in levels of IL-33 were detected in duodenum, ileum or for any of the time-points (Figures 4A–C,J,L).

The levels of the alarmin TSLP in duodenum were increased in the DON group after 4 h (Figures 4E,G). The TSLP levels showed no statistically significant differences in duodenum after 24 h (Figures 4F,H), or in ileum between any groups at any time point (Figures 4M–P). No clear effects of any of the compounds were observed for the soluble IL-33 receptor, ST2, for any time points in duodenum (data not shown). In ileum, the levels of ST2 were below the detection limit in both experiments.

A statistically significant difference was detected for IL-25 levels in duodenum between the DON + PN group and the HDM + PN group 48 h after exposure (data not shown). No other effects were detected for IL-25 in the intestines after exposure to any of the compounds tested (data not shown).

#### 3.2.2 Altered barrier function: ST2 in serum

**Figure 5:**
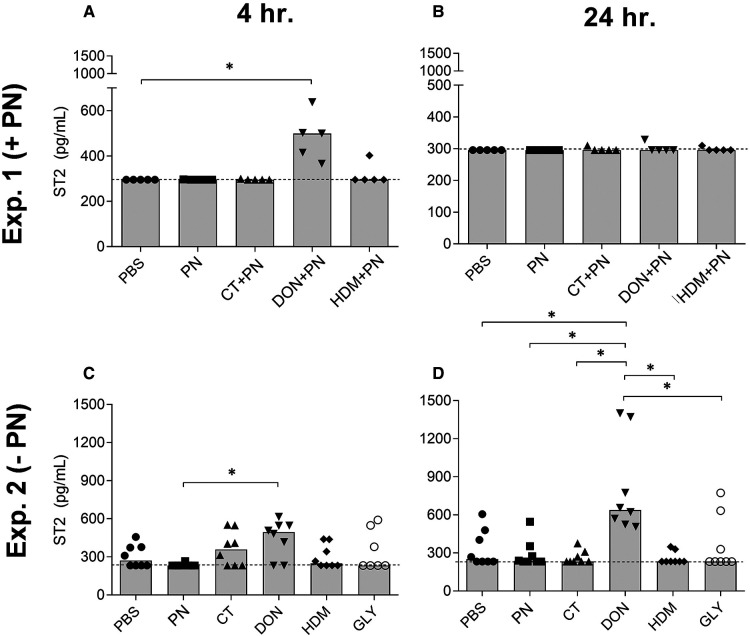
ST2 concentrations in serum at 4 h (**A**) and 24 h (**B**) after a single i.g. dose of PBS, PN or a barrier disruptor with PN (exp. 1, *n* = 5), 4 h (**C**) and 24 h (**D**) after a single i.g. dose of PBS, PN, or a barrier disruptor withour PN (exp. 2, *n* = 8). Dotted lines represent the lower assay detection limit. Brackets denote statistically significant differences (*p < 0.05, **p < 0.01) between two groups. Columns denote the group median value while each symbol denotes the individual animals.

Exposure to DON with PN resulted in statistically significantly increased levels of the IL-33 receptor ST2 in serum after 4, but not after 24 h (Figures 5A,B). ST2 levels after exposure to DON without PN were significantly increased both after 4 (only compared to the PN exposed group) and 24 h (all groups except HDM) (Figures 5C,D, respectively). No effects were seen for the other compounds tested, or for any components 48 h after exposure (data not shown).

#### 3.2.3 Gut barrier permeability: FABP2 and Ara h2 in serum

The serum levels of FABP2 were low (data not shown) and Ara h2 levels were below the detection limit in exp. 1. Thus, FABP2 and Ara h2 were not analyzed further in exp. 2.

#### 3.2.4 Lymphoid immune cell responses

**Figure 6:**
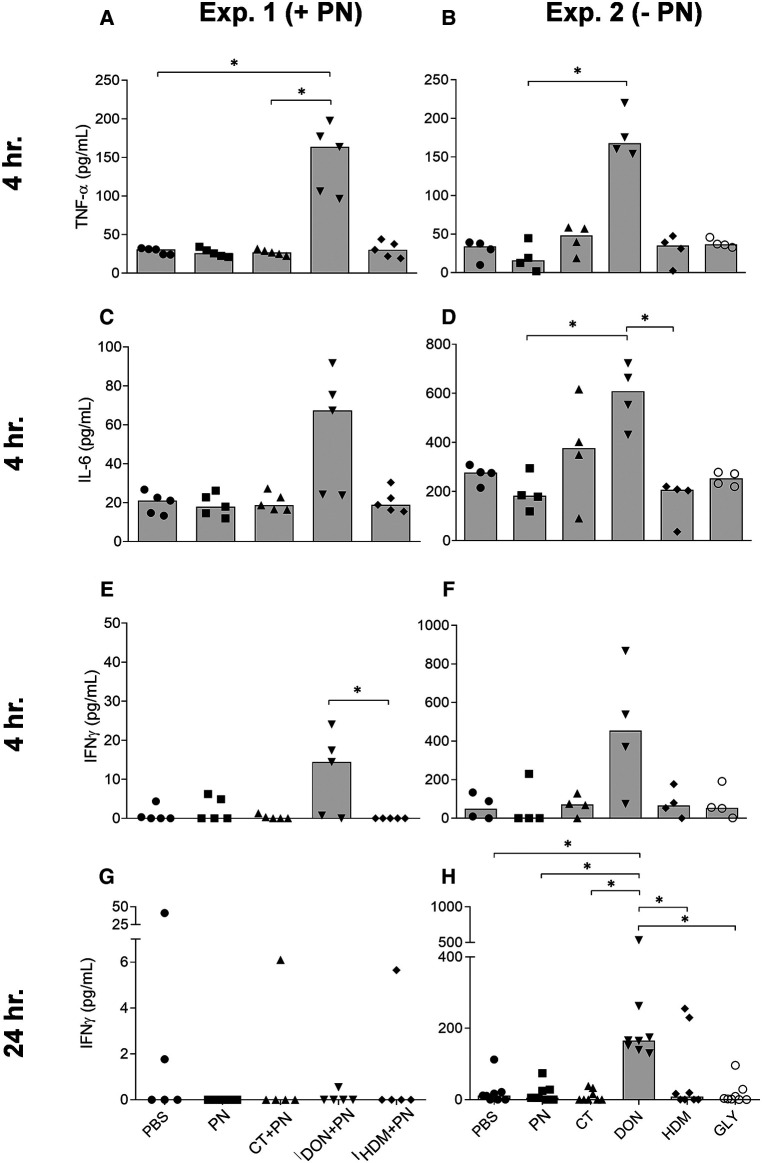
TNF-α, IL-6 and INFγ levels in MLN cells stimulated with LPS ex vivo, after a single i.g. dose of PBS, PN or a barrier disruptor with (exp. 1, *n* = 5) or without (exp. 2, *n* = 8) PN. The figures show levels of TNF-α (**A, B**), IL-6 (**C, D**), INFγ (**E, F**) 4 hours after exposure in exp. 1 (**A, C, E**) or exp. 2 (**B, D, F**). Also, levels of INFγ 24 h after exposure are shown for exp. 1 (**G**) and exp. 2 (**H**). Brackets denote statistically significant differences (*p < 0.05, **p < 0.01). Columns denote the group median value while each symbol denotes the individual animals.

In supernatants from LPS-stimulated mesenteric lymph node (MLN) cells, the cytokines TNF-α, IL-6, IFNγ and IL-1β were analyzed. The groups exposed to DON, both with or without PN, showed increased levels of TNF-α, IL-6 and IFNγ after 4 h in both experiments (Figures 6A–F).

IFNγ also showed a strong and statistically significant increase 24 h after DON exposure, but not after co-exposure with PN (Figures 6H,G). No statistically significant group differences were observed for TNF-α and IL-6 at the two other time-points in any of the two experiments, except a trend for elevated TNF-α levels after 48 h (Kruskal-Wallis test *p*-value of 0.0159, data not shown). The levels of IL-1β were below the detection limit in exp. 1 and low in exp. 2, but the group exposed to DON showed statistically significantly higher levels of IL-1β 4 h after exposure (data not shown).

The cytokine concentrations in supernatants from LPS-stimulated Peyer' patches (PP) cells (assessed only in exp. 2) 24 h after exposure were low and fluctuated close to the detection limit for all four cytokines. No statistically significant group differences were observed (data not shown).

In supernatants from ConA-stimulated MLN cells, the cytokines IL-5 and 13 were analyzed in both experiments, as well as IL-10, −17 and IFNγ in exp. 1 and IL-1β, IL-2, IL-4, and IL-6 in exp. 2. With two exceptions, no statistically significant group differences were seen at any time point in any of the experiments (data not shown). The exceptions were an overall significant increase in the levels of IL-5 at 24 h after exposure, and IL-17 at 48 h after exposure (*p*-values of 0.0245 and 0.0374 from the Kruskal–Wallis test, respectively). No statistically significant pairwise group differences were found. The levels of IL-4 and IL-1β were both below the assay detection limit.

## 4 Discussion

By assessing effects of known and suspected barrier disruptors, we found that CT and DON exerted an adjuvant effect on development of peanut allergy, while HDM and GLY did not show convincing effects at the doses tested. Although less clear than the positive control CT + PN, increased anaphylactic score and a trend of increased levels of total IgE in the DON + PN group indicate that oral exposure to DON promoted development of allergy in our model. This is supported by the finding that oral DON exposure acted as an adjuvant also in a mouse model of whey allergy (7). Our results suggest that CT was a stronger adjuvant than DON, since doses of CT were much lower (15 µg CT vs. 100 µg of DON), also in agreement with the whey allergy model (7).

Regarding early effects, the most prominent results were seen in the groups exposed to DON, with or without co-exposure to the PN allergen. Levels of the alarmins IL-33 and TSLP in intestinal tissue, the IL-33 receptor ST2 in serum, and the immune response markers TNF-α, IL-6 and IFNγ released from LPS-stimulated MLN cells were all increased in DON-exposed groups 4 or 24 h after a single exposure, in both short-term experiments. Few of these markers were convincingly affected after a single CT exposure, and HDM and GLY did not clearly affect any of these markers. In summary, while both DON and CT promoted development of clinical anaphylaxis in the peanut food allergy model, only DON showed a clear pattern of effects on the early markers (short-term experiments). Thus, the present study indicates that the underlying mechanisms of adjuvanticity of CT and DON differs. However, this did not support our working hypothesis that the chosen early markers could identify the adjuvants promoting development of food allergy in the model. Lack of responses of early markers were however associated with limited food allergy responses for HDM and GLY. Various mechanisms leading to adjuvanticity and thus promoting food allergy, however, may point to the need for assessing a broad set of markers capturing various pathways and/or high dimensional immune profiling, to enable prediction of compounds acting as allergy adjuvants.

Considering the different short-term effects after exposure to DON and CT, it is of interest to note that the two groups also responded differently after the PN challenge dose *in vivo*. Strong and weak effects of DON and CT, respectively, on intestinal alarmin production were also reported in the whey allergy model (7). They also reported that CT most strongly affected other early markers such as upregulation of costimulatory molecules and MHC class II in MLN cells, supporting our observations suggesting different mechanistic pathways for the adjuvant activity of DON and CT.

As alarmins rise in response to damaged epithelial cells (9, 10), it could be anticipated that the levels of alarmins in the intestine would rise early after exposure to barrier disruptors and drive pro-allergenic responses. In both short-term experiments, the intestinal levels of IL-33, but not IL-25 or ST2, were increased after DON exposure. Together with the simultaneous increase in serum levels of ST2, this demonstrate that DON exert early effects on the IL-33 pathway. TSLP levels were weakly (non-significantly) but consistently increased in both experiments 4 h after DON exposure. Our findings are in agreement with earlier studies suggesting that IL-33 play a central role, while TSLP seem to be associated with, but not playing an essential part, in allergen sensitization (44, 45).

MLN cells were stimulated with ConA or LPS to activate T cells or B cells and monocytes, respectively. ConA-induced cytokine release from MLN cells were not affected by treatment with any of the compounds. This may be due to the short time periods studied after exposure (4–48 h), as lymph node responses in other allergy models have been shown to be at its maximum 5–8 days after immunization with an allergen together with an adjuvant (46). However, we observed that MLNs stimulated with LPS were more sensitive to reflect responses at these early time points, and induced strong TNF-α, IL-6 and IFNγ production in cells from DON-exposed mice. Inflammatory cytokines may promote allergic sensitization, as demonstrated for TNF-α, acting as an allergy adjuvant in airway models (47). Taken together with the observed increase of alarmins in the intestines after exposure to DON and previous reports of DON-induced inflammation in the intestines (48), this suggests that early-stage inflammation may have contributed to the observed adjuvant effect of DON on food allergy development.

Exposure to HDM or GLY did not show adjuvant effects on food allergy development or effects on the early markers in the present experiments. However, HDM allergens have demonstrated allergy-promoting capacities for airway allergic disease (21, 44, 49). To our knowledge, we are the first to investigate HDM adjuvant potential after oral exposure, inspired by the observations of HDM presence in human gut and breast milk and Der p1s capacity of epithelial barrier disruption (19, 50). Our data showed that two of the twelve mice exposed to HDM + PN had high anaphylactic scores after PN challenge. One of these mice also had strongly elevated mMCP-1 levels in serum and a dramatic drop in body temperature, while the other had a strongly increased production of PN-induced inflammatory cytokines TNF-α, IL-6 and IFNγ. No significant effects were, however, detected on group level, although the group median value for the anaphylactic score seemed to be somewhat increased. We know from the present and previous studies in food allergy mouse models, that high and low responders exist, even within positive control groups receiving CT with allergen (7, 33, 51, 52). Furthermore, in this explorative study we did only include one dose of HDM, and we cannot exclude that higher doses could have revealed a higher number of responders. Our data raise concern that HDM have the capacity to act as an allergy adjuvant after oral exposure, although the adjuvant capacity of HDM in intestine and food allergy development remains speculative. Further investigations covering a dose-range of HDM in the gut is encouraged.

As Ara h2-IgE levels were below the detection limit for most animals and PN-specific IgE could not be detected due to methodological challenges (see methods section), total IgE was the only parameter reflecting sensitization in the present experiment. Systemic anaphylaxis can be mediated by crosslinking of IgE on mast cells or by IgG antibodies activating low-affinity IgG receptor FcgRIII, and macrophages (53–55). Although a (classic) IgE-driven pathway was the initial theory for the mechanisms behind the clinical anaphylaxis in our model (6), the lack of a proper measure for IgE sensitisation means that we cannot know from the present results whether the anaphylactic response in the group given DON with the allergen is mediated by IgG og IgE-driven mechanisms. In our food allergy model, the CT and PN exposed group had a clear increase in mMCP-1 serum levels, which indicates an IgE-mediated anaphylactic response (6). For the DON and PN exposed group, a clear mMCP-1 response was not detected. This could possibly point in the direction of DON mediating an IgG-response, rather than and IgE, as anti-FcγRII/RIII mAb failed to induce any increase in MMCP-1 (53). However, the food allergy model used have previously been shown to induce allergen-specific IgE as well as anaphylactic responses after allergen challenge (6), supportive of similar mechanisms in the current study.

In addition to the effects on the total IgE in the CT + PN and DON + PN groups, we also observed a trend of elevated levels in the group exposed to CT alone. This may suggest that the CT exposed mice had become sensitized to proteins such as soy, wheat, or barley in their feed, as this was the only source of food proteins in the group exposed to CT without PN. This is supported by the weak but statistically significantly elevated serum levels of mMCP-1 and a tendency of a temperature-drop in this group. A cross-reaction between sensitization to soy in the feed and PN as the challenging food allergen may have contributed to these observations (35, 56, 57).

Considerations such as reducing the number of animals, and practicalities such as workload, influenced the experimental design. Thus, screening several markers of endpoints such as intestinal permeability, alarmin production and local immune responses, were favored over choosing to administer more than one dose of the barrier disruptors. Consequently, lack of different doses of the barrier disruptors is one obvious limitation of the present study.

In conclusion, among the known and suspected barrier disrupting compounds and doses tested in the present mouse model, CT and DON acted as adjuvants by promoting development of peanut food allergy. DON was the only barrier disrupting compound clearly activating alarmins in the intestines. As both DON and CT were found to promote clinical food allergy, but the investigated early markers were only clearly affected by DON, our results suggest that DON and CT have different modes of action at the early stages of sensitization. Our results add to the accumulating evidence of detrimental immune effects of DON, which is of particular concern based on its exposure levels reported to exceed current Tolerable Daily Intake in children, with increasing challenges with climate changes (18, 58). To further challenge the field of the “barrier disruption hypothesis” in the light of the increasing prevalence of food allergies worldwide, we recommend that a combination of a wider set of dose-ranges, time-points and markers reflecting different modes of action is necessary to predict compounds with food allergy adjuvant capacity.

## Data Availability

The raw data supporting the conclusions of this article will be made available by the authors, without undue reservation.
